# Molecular genetic divergence analysis amongst high curcumin lines of Golden Crop (*Curcuma longa* L.) using SSR marker and use in trait-specific breeding

**DOI:** 10.1038/s41598-023-46779-5

**Published:** 2023-11-11

**Authors:** Anindita Gogoi, Sunita Munda, Manabi Paw, Twahira Begum, Manzer H. Siddiqui, Abdel-Rhman Z. Gaafar, Mahipal Singh Kesawat, Mohan Lal

**Affiliations:** 1 Academy of Scientific and Industrial Research, Ghaziabad, UP 201002 India; 2 CSIR-North East Institute of Science and Technology, Jorhat, Assam, 785006 India; 3 Department of Botany and Microbiology, College of Science, King Saud University, Riyadh, 11451 Saudi Arabia; 4 Institute for Molecular Biology and Genetics, School of Biological Sciences, Seoul National University, Seoul, 08826 South Korea

**Keywords:** Genetics, Molecular biology

## Abstract

*Curcuma longa* L., is recognized worldwide as a medicinally and economically important plant species due to its curcumin content which is an industrially important compound. In this study, a total of 329 accessions were collected from four states of India and planted in the experimental farm of CSIR-NEIST, Jorhat, India, in augmented design. Among these, 152 high curcumin (> 1.50%) accessions were screened for molecular divergence study using 39 SSR primers. The primers showed the most efficient outcome with 2–8 allele/ loci and a total 163 number of alleles with 100% polymorphism. Cluster analysis revealed the construction of three clusters, out of which one cluster was geographically dependent, and germplasm was particularly from Assam state. Jaccard's pairwise coefficient showed maximum genetic dissimilarity of (0.75) between accession RRLJCL 3 and RRLJCL 126, indicating high variation as it was from two different states viz Arunachal Pradesh and Nagaland respectively and minimum genetic dissimilarity of (0.09) between RRLJCL 58 and RRLJCL 59 indicating significantly less variation as the two accessions were from same state, i.e., Arunachal Pradesh. Analysis of Molecular Variance (AMOVA) revealed high molecular variation within the population (87%) and significantly less variation among the population (13%). Additionally, Neighbour Joining dendrogram, Principal Component Analysis (PCA), and bar plot structure revealed similar clustering of germplasm. This diversity assessment will help in selecting the trait-specific genotypes, crop improvement program, conservation of gene pool, marker-assisted breeding, and quantitative trait loci identification. Moreover, to the best of our knowledge, it is the first molecular diversity report among 152 high curcumin lines of *C. longa* from North East India using 39 SSR primers.

## Introduction

In the present era of great scientific studies, acknowledging the importance of medicinal crops and shifting towards therapeutic agents is increasing tremendously. Among all, the genus Curcuma, which belongs to the Zingiberaceae family, is acknowledged medicinally and economically as one of the most predominate plant species^1,2^.*Curcuma longa* L. generally known as turmeric, is widely distributed throughout South and South East Asia, with the most diversity concentrated in Thailand and India, followed by Bangladesh, Vietnam, Myanmar, and Indonesia^3^. It is an interbreed triploid species (3n = 63) which is procreate vegetatively using the below-ground rhizome^4^. Morphologically, turmeric is a perennial herb that measures up to 1 m in height with a wee stem, having funnel-shaped yellow flowers and oblong, pointed leaves^5^.

Since time immemorial, turmeric as flavoring spice powder has been used continuously in the preparation of both vegetarian and non-vegetarian food due to its digestive properties^6^. In addition to its uses in religious practices, the rhizome part of *C. longa* has numerous masteries in traditional medicines like stimulants, diabetic wounds, jaundice, to treat liver complaints, stomachic, arthritic, muscular disorders, blood purifiers, rheumatism, cough, and sinusitis like diseases^7,8^. The *C. longa* rhizomes are also widely practiced in treating sprains and swelling caused due to different injuries^9^. The primary compound of turmeric is curcuminoid which is the most active component, and it prevails in three polyphenolic forms, namely curcumin, demethoxycurcumin, and bisdemethoxycurcumin^10–12^. The chemical compounds like curcumin (1,7-bis(4-hydroxy-3-methoxyphenyl)-1,6-heptadiene-3,5-dione), which is also better known as diferuloylmethane is one of the key natural polyphenols found in the turmeric rhizome. It exists in two tautomeric forms: enolic in organic solvents and keto in water^13^. Curcumin is the foremost essential component present in the *C. longa* rhizomes, which is responsible for the yellow hue. It is responsible for several pharmacological activities like anti-inflammatory, anti-hepatotoxic, anti-microbial, anti-rheumatic, anti-fibrotic, choleretic, anti-venomous, hypercholesteremic, anti-diabetic, insect repellent as well as anti-cancerous properties^4,7,14^.

Additionally, the essential oil (EO) of *C. longa*, which is extracted from the leaf, flower, root, and rhizome of the plant, is considered as a highly valuable product in different pharmaceutical and cosmetic industries. The essential oil (EO) is epitomized by the existence of secondary metabolites, which possess a strong odour, volatile terpene, and hydrocarbon^15^. The chemical composition present in the EO of plants can vary due to various factors like nutrients present in the soil, temperature, humidity, altitude, ultraviolet radiation, luminosity, seasonality, circadian cycle, and portion of the plant^16–18^. The prime essential oil constituents of *Curcuma longa* consist of monoterpenoids and sesquiterpenoids^19^. Earlier studies outlined that in *C. longa*, rhizome essential oil comprised of constituents like ar-turmerone, turmerone, curlone, ar-curcumene, and root consists of ar-turmerone, dihydro curcumene, and ar-curcumene where components like turmerone were absent. Moreover, the major constituents found in leaf oil were* p-*cymene, terpinolene, α-phellandrene, 1,8- cineole, and flower oil consist of terpinolene,* p*-cymen-8-ol, 1,8-cineole^20^. These significant constituents were reported to exhibit numerous health benefits, which help to strengthen the immune system, expedite toxin elimination, and elevate blood circulation^21–23^.

High rhizome yield, disease resistance, and high curcumin content are the desirable characteristics for the turmeric varietal developmental programme, and North East India being a biodiversity hotspot, has ample variability of *C. longa* L. which needs a meticulous study. For screening of the elite germplasm, the study on genetic diversity as well as their association study is of immense significance. Though the value of this golden crop has been recognized since ages, but the demand for this crop in the domain of the food and pharmaceutical industry is still increasing^24–26^. Therefore, genetic diversity analysis was performed in order to enhance a deeper study in the productivity and conservation of this crop which forms the basis of the crop breeding program^27^. The application of phenotypic attribute in germplasm conservation is meagred due to the influence of environment and genotype interactions^28^; therefore, studies have been performed on molecular diversity using various markers like Simple Sequence Repeat (SSR), Randomly Amplified Polymorphic DNA (RAPD), Amplified Fragment Length Polymorphism (AFLP). The application of RAPD provides insightful information about genetic diversity^29^ but yields a low level of polymorphism as relative to SSR markers^30^. Molecular diversity was successfully studied in many crops for phylogenetic assessment and genetic diversity using SSR marker^31–34^ as SSR markers are locus-specific, co-dominant markers and have the capability to perceive high levels of miscellany in the genome^34–36^.

The present study was designed to estimate the genetic divergence of *C. longa* germplasm using a large number of accessions (152) gathered from different regions of North East India using SSR markers. A very few reports have been found where Singh et al.^37^ evaluated 30 genotypes of *C. longa* using 9 SSR markers, Sahoo et al.^38^ studied 88 accessions using 50 EST SSR primers, out of which only 11 primers showed polymorphic banding patterns. Till now, to our supreme cognizance, no molecular diversity survey has been performed on such a large number of accessions, and hence this study will help in the progress of worthier germplasm with more yield-related traits and crossing of parents with preferable traits^34^. This will provide more reliable information in improving traits and will be very effective for the selection and conservation of trait-specific germplasm, which can be further implemented in the study of the food and pharmaceutical industry.

## Results

### Selection of high curcumin lines

Among the 329 accessions collected from four states of India, 152 accessions were selected based on curcumin content (> 1.50%) for further molecular analysis. The total curcuminoids content of selected lines ranged between 1.86 and 12.97%, which is charted in (Table 1).

**Table 1:** Collection site of 152 germplasm along with their curcuminoid content in percentage.

S1. no	Accession number	Collection site	Curcuminoids content (%)
1	RRLJCL 1	Gulimora, Dibrugarh, Assam	6.86
2	RRLJCL 2	Kanchangjangha,Tinsukia, Assam	9.38
3	RRLJCL 3	Itanagar, Arunachal Pradesh	9.16
4	RRLJCL 4	Joporamukh, Dibrugarh, Assam	4.40
5	RRLJCL 5	Joporagaon, Dibrugarh, Assam	12.08
6	RRLJCL 6	Nijmankotagaon, Dibrugarh, Assam	2.71
7	RRLJCL 7	Motok Tea Estate, Dibrugarh, Assam	7.52
8	RRLJCL 8	Nagazanka, Jorhat,Assam	6.44
9	RRLJCL 9	Pasighat, East Siang, Arunachal Pradesh	4.46
10	RRLJCL 10	Dainijan, Dibrugarh,Assam	8.10
11	RRLJCL 11	Butolikhua Tea Estate, Golaghat, Assam	8.70
12	RRLJCL 12	Dainijan, Dibrugarh, Assam	1.86
13	RRLJCL 13	Mainbari Tea Estate, Dibrugarh, Assam	12.97
14	RRLJCL 14	ShawsanJopora Gaon, Dibrugarh, Assam	8.43
15	RRLJCL 15	Charaideo, Charaideo, Assam	5.68
16	RRLJCL 16	Pasighat, East Siang, Arunachal Pradesh	12.85
17	RRLJCL 17	Borboruah Tea Estate, Dibrugarh, Assam	4.17
18	RRLJCL 18	Khirpan, Nagaland	4.56
19	RRLJCL 19	Pasighat, East Siang, Arunachal Pradesh	2.49
20	RRLJ CL 20	Itanagar, Itanagar, Arunachal Pradesh	12.32
21	RRLJCL 21	July Basti, Itanagar, Arunachal Pradesh	10.5
22	RRLJCL 22	Itanagar, Itanagar, Arunachal Pradesh	12.19
23	RRLJCL 23	Longthe, Nagaland	8.91
24	RRLJCL 24	Itanagar, Arunachal Pradesh	4.28
25	RRLJCL 25	Ahubari Tea Estate, Dibrugarh, Assam	2.41
26	RRLJCL 26	Kanchanjongha Tea Estate, Tinsukia, Assam	6.54
27	RRLJCL 27	Nijmankota Gaon, Dibrugarh, Assam	11.30
28	RRLJCL 28	Jhanji, Sivasagar, Assam	7.37
29	RRLJCL 29	Jamuguri, Darrang, Assam	5.62
30	RRLJCL 30	Jhanji, Sivasagar,Assam	9.30
31	RRLJCL 31	Longthe, Nagaland	3.26
32	RRLJCL32	Sonari, Sivasagar, Assam	8.78
33	RRLJCL 33	Sepon, Sivasagar,Assam	8.78
34	RRLJCL 34	Sepon, Sivasagar, Assam	10.30
35	RRLJCL 35	Geleki, Sivasagar, Assam	7.72
36	RRLJCL 36	Hautely Tea Estate, Golaghat, Assam	4.89
37	RRLJCL 37	Itanagar, Arunachal Pradesh	4.44
38	RRLJCL 38	Daily market, Mon, Nagaland	11.32
39	RRLJCL 39	Wokha, Nagaland	7.87
40	RRLJCL 40	Pasighat, East Siang, Arunachal Pradesh	6.28
41	RRLJCL 41	Nagazanka, Jorhat, Assam	4.57
42	RRLJCL 42	Sonari, Sivasagar, Assam	10.62
43	RRLJCL 43	Dimapur, Nagaland	6.59
44	RRLJCL 44	Buralikson, Golaghat, Assam	7.74
45	RRLJCL 45	Dimapur, Nagaland	5.68
46	RRLJCL 46	Naga Village, Nagaland	9.93
47	RRLJCL 47	Ahubari tea state, Dibrugarh, Assam	8.97
48	RRLJCL 48	Sepon, Sivasagar, Assam	12.40
49	RRLJCL 49	Sepon, Sivasagar, Assam	9.45
50	RRLJCL 50	Nguvihe village, Nagaland	12.80
51	RRLJCL 51	Daily market, Mon, Nagaland	11.51
52	RRLJCL 52	Sonari, Sivasagar, Assam	5.28
53	RRLJCL 53	Pasighat, East Siang, Arunachal Pradesh	7.04
54	RRLJCL 54	Pasighat, East Siang, Arunachal Pradesh	12.94
55	RRLJCL 55	Pasighat, East Siang, Arunachal Pradesh	7.16
56	RRLJCL 56	Itanagar, Arunachal Pradesh	11.07
57	RRLJCL 57	Itanagar, Arunachal Pradesh	1.97
58	RRLJCL 58	Itanagar, Arunachal Pradesh	12.56
59	RRLJCL 59	Near Shiv Mandir, Itanagar, Arunachal Pradesh	5.7
60	RRLJCL 60	Itanagar, Arunachal Pradesh	3.32
61	RRLJCL 61	Pasighat, East Siang, Arunachal Pradesh	10.56
62	RRLJCL 62	Itanagar, Arunachal Pradesh	12.76
63	RRLJCL 63	Ziro, Lower Subansiri, Arunachal Pradesh	4.45
64	RRLJCL 64	Ziro, Lower Subansiri, Arunachal Pradesh	7.29
65	RRLJCL 65	Old Ziro, Lower Subansiri, Arunachal Pradesh	12.95
66	RRLJCL 66	Ramkrishna Mission, Aalo, West Siang, Arunachal Pradesh	3.78
67	RRLJCL 67	Itanagar, Arunachal Pradesh	4.6
68	RRLJCL 68	Pasighat, East Siang, Arunachal Pradesh	3.98
69	RRLJCL 69	Pasighat, East Siang, Arunachal Pradesh	4.4
70	RRLJCL 70	Motok Tea Estate, Dibrugarh, Assam	12.42
71	RRLJCL 71	Borpathar, Golaghat, Assam	9.01
72	RRLJCL 72	Dapi, East Siang, Arunachal Pradesh	9.02
73	RRLJCL 73	Dimapur, Nagaland	12.45
74	RRLJCL 74	PotiaPothar, Golaghat, Assam	12.39
75	RRLCL 75	Kachupathar, Golaghat, Assam	9.65
76	RRLJCL 76	Dhekial, Golaghat, Assam	10.91
77	RRLJCL 77	Pasighat, East Siang, Arunachal Pradesh	11.7
78	RRLJCL 78	Borpathar, Golaghat, Assam	4.40
79	RRLJCL 79	Itanagar, Arunachal Pradesh	8.7
80	RRLJCL 80	Gemon, Sivasagar, Assam	12.83
81	RRLJCL 81	Itanagar, Arunachal Pradesh	7.70
82	RRLJCL 82	Ramkrishna Mission, Aalo, West Siang, Arunachal Pradesh	5.4
83	RRLJCL 83	Ziro, Lower Subansiri, Arunachal Pradesh	6.28
84	RRLJCL 84	Nagaram Village, Manipur	11.31
85	RRLJCL 85	Dapi, East Siang,Arunachal Pradesh	3.40
86	RRLJCL 86	Pasighat, Itanagar, Arunachal Pradesh	9.58
87	RRLJCL 87	Near Shiv mandir, Itanagar, Arunachal Pradesh	9.37
88	RRLJCL 88	Nagaram Village, Manipur	12.28
89	RRLJCL 89	Nagaram Village, Manipur	2.84
90	RRLJCL 90	Itanagar, Arunachal Pradesh	10.94
91	RRLJCL 91	Nagaram Village, Manipur	8.85
92	RRLJCL 92	Pasighat, East Siang, Arunachal Pradesh	12.63
93	RRLJCL 93	Nagaram, Manipur	8.47
94	RRLJCL 94	Gemon, Sivasagar, Assam	12.74
95	RRLJCL 95	Sonari, Sivasagar, Assam	8.32
96	RRLJCL 96	Gauripur, Dhubri, Assam	10.67
97	RRLJCL 97	Charaideo, Charaideo, Assam	12.53
98	RRLJCL 98	Sonari, Sivasagar, Assam	11.55
99	RRLJCL 99	Sonari, Sivasagar, Assam	8.90
100	RRLJCL 100	Sonari, Sivasagar, Assam	9.8
101	RRLJCL 101	Gargaon, Sivasagar, Assam	8.14
102	RRLJCL 102	Gargaon, Sivasagar, Assam	10.21
103	RRLJCL 103	Sonari, Sivasagar, Assam	5.34
104	RRLJCL 104	Moran, Dibrugarh, Assam	7.21
105	RRLJCL 105	Morigaon, Assam	12.64
106	RRLJCL 106	Morigaon, Assam	9.71
107	RRLJCL 107	Gemon, Sivasagar, Assam	10.14
108	RRLJCL 108	Sonari, Sivasagar, Assam	6.26
109	RRLJCL 109	Khirpan, Nagaland	6.64
110	RRLJCL 110	Daily market, Mon, Nagaland	7.71
111	RRLJCL 111	Wokha,Nagaland	7.43
112	RRLJCL 112	Nagazanka, Jorhat, Assam	5.67
113	RRLJCL 113	Nagazanka, Jorhat, Assam	4.23
114	RRLJCL 114	Itanagar, Arunachal Pradesh	12.3
115	RRLJCL 115	Longso, Karbi Anglong, Assam	12.72
116	RRLJCL 116	Wokha, Nagaland	12.24
117	RRLJCL 117	Boko, Kamrup, Assam	9.10
118	RRLJCL 118	Daily market, Mon, Nagaland	6.46
119	RRLJCL 119	Pasighat, East Siang, Arunachal Pradesh	5.67
120	RRLJCL 120	Mohmora, Dibrugarh, Assam	6.64
121	RRLJCL 121	Khirpan, Nagaland	8.83
122	RRLJCL 122	Aalo, West Siang, Arunachal Pradesh	7.80
123	RRLJCL 123	Wokha, Nagaland	12.02
124	RRLJCL 124	Daily market, Mon, Nagaland	10.92
125	RRLJCL 125	Sonari, Sivasagar. Assam	6.3
126	RRLJCL 126	Gemon, Sivasagar, Assam	4.89
127	RRLJCL 127	Sonari, Sivasagar, Assam	4.67
128	RRLJCL 128	Sonari, Sivasagar, Assam	7.72
129	RRLJCL 129	Morigaon, Morigaon, Assam	3.44
130	RRLJCL 130	Shilani, Nagaon, Assam	4.10
131	RRLJCL 131	Selek, Dhemaji, Assam	7.86
132	RRLJCL 132	Dimapur, Nagaland	7.8
133	RRLJCL 133	Longsa, Wokha, Nagaland	8.23
134	RRLJCL 134	Dimapur, Nagaland	8.89
135	RRLJCL 135	Longtha, Nagaland	7.8
136	RRLJCL 136	Meshil, Nagaland	8.65
137	RRLJCL 137	Longsa,Wokha, Nagaland	7.8
138	RRLJCL 138	Garampani, Golaghat, Assam	8.48
139	RRLJCL 139	Naga Village, Nagaland	5.57
140	RRLJCL 140	Wokha, Nagaland	3.67
141	RRLJCL 141	Touphene village, Kohima, Nagaland	8.30
142	RRLJCL 142	Shilonijan, Karbi Anglong, Assam	9.90
143	RRLJCL 143	Garampani, Golaghat, Assam	5.41
144	RRLJCL 144	Merapani,Golaghat, Assam	6.77
145	RRLJCL 145	Shilonijan, Karbi Anglong, Assam	10.91
146	RRLJCL 146	Touphene village, Kohima, Nagaland	12.78
147	RRLJCL 147	Aalo, West Siang, Arunachal Pradesh	4.10
148	RRLJCL 148	Selek, Dhemaji, Assam	8.80
149	RRLJCL 149	Merapani,Golaghat Assam	12.6
150	RRLJCL 150	Liphi village, Wokha, Nagaland	9.10
151	RRLJCL 151	Bongaigaon. Assam	8.9
152	RRLJCL 152	Barpeta, Assam	6.67

### SSR primer competency

For screening of efficient primer, a total of 44 SSR Primers were identified based on review of previous literature on the family Zingiberaceae. Primer testing was executed using all 44 SSR primers by maintaining the melting temperatures of the selected primers, of which 39 primers showed efficient results like sound amplification, better reproducible pattern, number of polymorphic fragments per assay, and level of polymorphism detected. The primer sequences, polymorphism percentage, Polymorphism Information Centre (PIC), Resolving Power (Rp), and Marker index (MI) outcomes of the screened primers are depicted in Table 2.

**Table 2:** List of SSR primers used in the study along with their primer sequence, polymorphic allele, Polymorphic Information Content (PIC), Marker index (MI), and Resolving Power (Rp).

Sl. no	Primers	Primer sequence (5ʹ–3ʹ) (3ʹ–5ʹ)	Total allele	Polymorphic allele	% Polymorphism	PIC	MI	Rp
1	CuMiSat-19F CuMiSat-19R	CATGCAAATGGAAATTGACAC TGATAAATTGACACATGGCAGTC	4	4	100	0.71	2.84	1.86
2	CuMiSat-20F CuMiSat-20R	CGATACGAGTCCATCTCTTCG CCTTGCTTTGGTGGCTAGAG	2	2	100	0.25	0.5	1
3	CuMiSat-21F CuMiSat-21R	TCATTCAAAAGTCCGATGGAA TTCGAGTGCAGAAGGAATTA	3	3	100	0.72	2.16	1.52
4	CuMiSat-22F CuMiSat-22R	AATTTATTAGCCCGGACCAC AAGAAAGTGAGTAGAAACCAAAGC	8	8	100	0.79	6.32	1.82
5	CuMiSat-23F CuMiSat-23R	CGTGGAAGGTGAGTTTGAC CAGAAGGGAACTGAGATGG	2	2	100	0.23	0.46	0.92
6	CuMiSat-24F CuMiSat-24R	AGGTATTCTACTCGACCAAG AAATTCATATAGCCCCATC	2	2	100	0.50	1.01	0.34
7	CuMiSat-25F CuMiSat-25R	TACATGAGAAACAACAAAGCCC AGTTAGCCAAGTCCCAATTTAGC	2	2	100	0.46	0.93	1.86
8	CuMiSat-26F CuMiSat-26R	CATTCCGATGAATTGTATG GCAGTTGTTTTGCTTCAG	5	5	100	0.78	3.9	0.88
9	CuMiSat-27F CuMiSat-27R	TATAGATAGCCATGCTGAAG CCATTTTAGTTCATTACGTG	4	4	100	0.62	2.48	0.94
10	CuMiSat-28F CuMiSat-28R	TTCAACTTCTCCTCGCTCAG GCAAGGTCTGCATCTATTTCTC	2	2	100	0.70	1.4	1.22
11	CuMiSat-30F CuMiSat-30R	CTCTAATGTCGCCTCTCACG GCATCTCCCGTTCTTCTCC	3	3	100	0.46	1.39	1.1
12	CuMiSat-31F CuMiSat-31R	GGAGGAGAAGAAGCAGAAG GACAGGCGAAGGAGGAAAC	6	6	100	0.89	5.34	1.34
13	CuMiSat-32F CuMiSat-32R	TGTTGTAGGTAGAAGCAAATGAC TTGGTGTCCAATTTCTTTCAAC	2	2	100	0.47	0.94	0.32
14	CuMiSat-33F CuMiSat-33R	ATGGATGGATACAACAACAACAAC TATAAACACACTCCCTCTTGG	3	3	100	0.58	1.74	2.08
15	CuMiSat-34F CuMiSat-34R	AAGTTGGTGAAGGATTAGAGCTAC CACCTAGTGGGATAAATCTTGG	3	3	100	0.60	1.8	1.52
16	CuMiSat-35F CuMiSat-35R	GGTTCGTCGTGGAAAGTAAT GCATCTCAACAGGGGCTG	3	3	100	0.75	2.25	1.18
17	CuMiSat-36F CuMiSat-36R	TGGGCTCAATGGTTGATACG CTCCTCATCGCTATCCGAGG	2	2	100	0.28	0.57	1.14
18	CuMiSat-37F CuMiSat-37R	CCATTGGCGAGGATGAAGC CCTGCCAAGCAAAGCCAAG	6	6	100	0.62	3.72	3.46
19	CuMiSat-38F CuMiSat-38R	TCATCATAAACACTCCTCTG GAAGAAGAGGCTAAGTTC	7	7	100	0.86	6.02	0.12
20	CuMiSat-39F CuMiSat-39R	TATCCCCTGAAAAACTAATCC AAAATGTCACGAACTATTGC	2	2	100	0.68	1.36	1.26
21	RM117F RM117R	CGATCCATTCCTGCTGCTCGCG CGCCCCATGCATGAGAAGACG	5	5	100	0.77	3.85	0.48
22	RM125F RM125R	ATCAGCAGCCATGGCAGCGACC AGGGGATCATGTGCCGAAGGCC	7	7	100	0.77	5.39	2.46
23	RM131F RM131R	TCCTCCCTCCCTTCGCCCACTG CGATGTTCGCCATGGCTGCTCC	3	3	100	0.87	2.61	0.8
24	RM135F RM135R	CTCTGTCTCCTCCCCCGCCTCCTC TCAGCTTCTGGCCGGCCTCCTC	5	5	100	0.67	3.36	1.52
25	RM153F RM153R	GCCTCGAGCATCATCATCAG ATCAACCTGCACTTGCCTGG	4	4	100	0.71	2.85	1.22
26	RM154F RM154R	ACCCTCTCCGCCTCGCCTCCTC CTCCTCCTCCTGCGACCGCTCC	5	5	100	0.71	3.56	1.24
27	RM171F RM171R	AACGCGAGGACACGTACTTAC ACGAGATACGTACGCCTTTG	2	2	100	0.5	1	0
28	RM278F RM278R	GTAGTGAGCCTAACAATAATA TCAACTCAGCATCTCTGTCC	6	6	100	0.83	5.01	0.02
29	RM287F RM287R	TTCCCTGTTAAGAGAGAAATC GTGTATTTGGTGAAAGCAAC	7	7	100	0.84	5.88	0.32
30	CBT-02F CBT-02R	TCCTCCTCCCTTCGCCCACTG CGATGTTCGCCATGGTGCTCC	4	4	100	0.73	2.92	0.18
31	CBT-03F CBT-03R	ATCAGCAGCCATGGCAGCGAC AGGGGATCATGTGCCGAAGGC	5	5	100	0.55	2.76	2.56
32	CBT-04F CBT-04R	ACCCTCTCCGCCTCGCCTCCTC CTCCTCCTCCTGCGACCGCTCC	5	5	100	0.58	2.89	2.98
33	CBT-05F CBT-05R	CTCTGTCTCCTCCCCGCGTCG TCAGCTTCTGGCCGGCCTCCTC	6	6	100	0.82	4.92	1.8
34	CBT-07F CBT-07R	CGATCCATTCCTGCTGCTCGCG CGCCCCCATGCATGAGAAGAG	7	7	100	0.85	5.96	0.6
35	CBT-08F CBT-08R	CAGCAGATTTTTGCTCCG GTCGCGTTCGTGGAAAT	6	6	100	0.75	4.51	1.42
36	MAGN27F MAGN27R	CATGTATTGGGCAGATATACATG TTCGACACATAGTTCCTGAAAC	2	2	100	0.20	0.4	0.8
37	MAGN78F MAGN78R	ATGTTCCACTTATCCTTTCAG CGATATCAAGAGATCAAAGG	3	3	100	0.74	2.23	1.54
38	HM469957F HM469957R	ATGTTCCACTTATCCTTTCAG CGATATCAAGAGATCAAAAGG	2	2	100	0.40	0.8	0.72
39	HM469963F HM469963R	ATAAACAACAACTAGCTCTTAG TAAATCTATAGGAACCTTAG	3	3	100	0.63	1.89	0.42
Total			163	163	3900	24.87	109.92	46.96
Average			3.97	3.97	100	0.64	2.82	1.204
Standard deviation			1.85	1.85	0	0.18	1.78	0.80

The screened 39 primers showed good amplified bands, which ranged between 2 and 8 alleles per individual in all the loci. A total of 163 alleles were achieved, of which all (163) were polymorphic, and no monomorphic bands were observed. The polymorphism percentage of all the primers showed 100%, which is unique to this study. Furthermore, PIC, Rp, and MI values were evaluated to check the proficiency of the screened primers. In the current survey, the PIC value of all primers ranged between 0.20 and 0.89, of which primer (MAGN27) showed minimum PIC value and primer (CuMiSat 31) showed maximum PIC value. The primer proficiency was further analyzed by calculating the Marker Index (MI) and Resolving Power (Rp). The average MI was estimated to be 2.82, of which primer (MAGN27) showed a minimum of 0.40, and CuMiSat 22 showed a maximum of 6.32. Similarly, the Rp value was also evaluated where Rp is the resolving power which objective is to discriminate between genotypes, and from the calculated study, it was observed that the average Rp value was 1.204, of which primer (RM 171) showed a minimum Rp value (0) and primer (CuMiSat 37) showed maximum Rp value (3.46). The gel image of the SSR primer (CuMiSat-37) profile of all the 152 accessions of *C. longa* is represented in Fig. S1.

### Genetic diversity of inter and intra population

The genotypes of selected lines were splitted into four populations, namely Pop 1 from Assam, Pop 2 from Arunachal Pradesh, Pop 3 from Nagaland, and Pop 4 from Manipur, based on the geographical location. The genetic variability parameters like the number of observed alleles (na), Nei’s gene diversity (h), number of effective alleles (ne), Shannon’s information index (I), genetic diversity within the population (Hs), genetic differentiation degree (Gst), genetic diversity in the population (Ht), gene flow (Nm) are computed and mentioned in Table 3. The parameters (na, ne, h, I) showed highest in Population 1 of Assam (1.93, 1.42, 0.26, 0.40), followed by Population 2 of Arunachal Pradesh (1.89, 1.43. 0.26, 0.39), Population 3 of Nagaland (1.76, 1.41, 0.24, 0.36) and lowest from Population 4 of Manipur (1.50, 1.30, 0.17, 0.26). Moreover, significant genetic diversity was confirmed from the result of total species diversity within the population (Ht) and among the population (Hs): 0.27 ± 0.03 and 0.23 ± 0.02 respectively, with genetic differentiation degree (Gst) of 1.24 and gene flow (Nm) of 3.51 which is significantly higher.

**Table 3:** Genetic diversity of *C longa* collected from four different states (Assam, Arunachal Pradesh, Nagaland, Manipur) using following parameters (number of observed alleles (na), number of effective alleles (ne), Nei’s gene diversity (h), Shannon's information index (I), genetic diversity in the population (Ht), genetic diversity within the population (Hs), genetic differentiation degree (Gst), gene flow (Nm).

Sl. no	Population	Mean				Polymorphic %	Ht	Hs	Gst	Nm
		na	ne	h	I					
1	(Pop 1) Assam	1.92 ± 0.26	1.42 ± 0.33	0.26 ± 0.17	0.40 ± 0.22	92.41	0.27 ± 0.03	0.23 ± 0.02	1.24	3.51
2	(Pop 2) Arunachal Pradesh	1.89 ± 0.31	1.43 ± 0.37	0.25 ± 0.18	0.39 ± 0.24	88.61				
3	(Pop 3) Nagaland	1.76 ± 0.42	1.4 ± 0.38	0.24 ± 0.19	0.36 ± 0.26	75.95				
4	(Pop 4) Manipur	1.50 ± 0.50	1.30 ± 0.36	0.17 ± 0.19	0.26 ± 0.28	50				
Average		1.99	1.45	0.27	0.41	98.73				
Standard deviation		0.11	0.35	0.17	0.22					

### Cluster analysis

**Figure 1:**
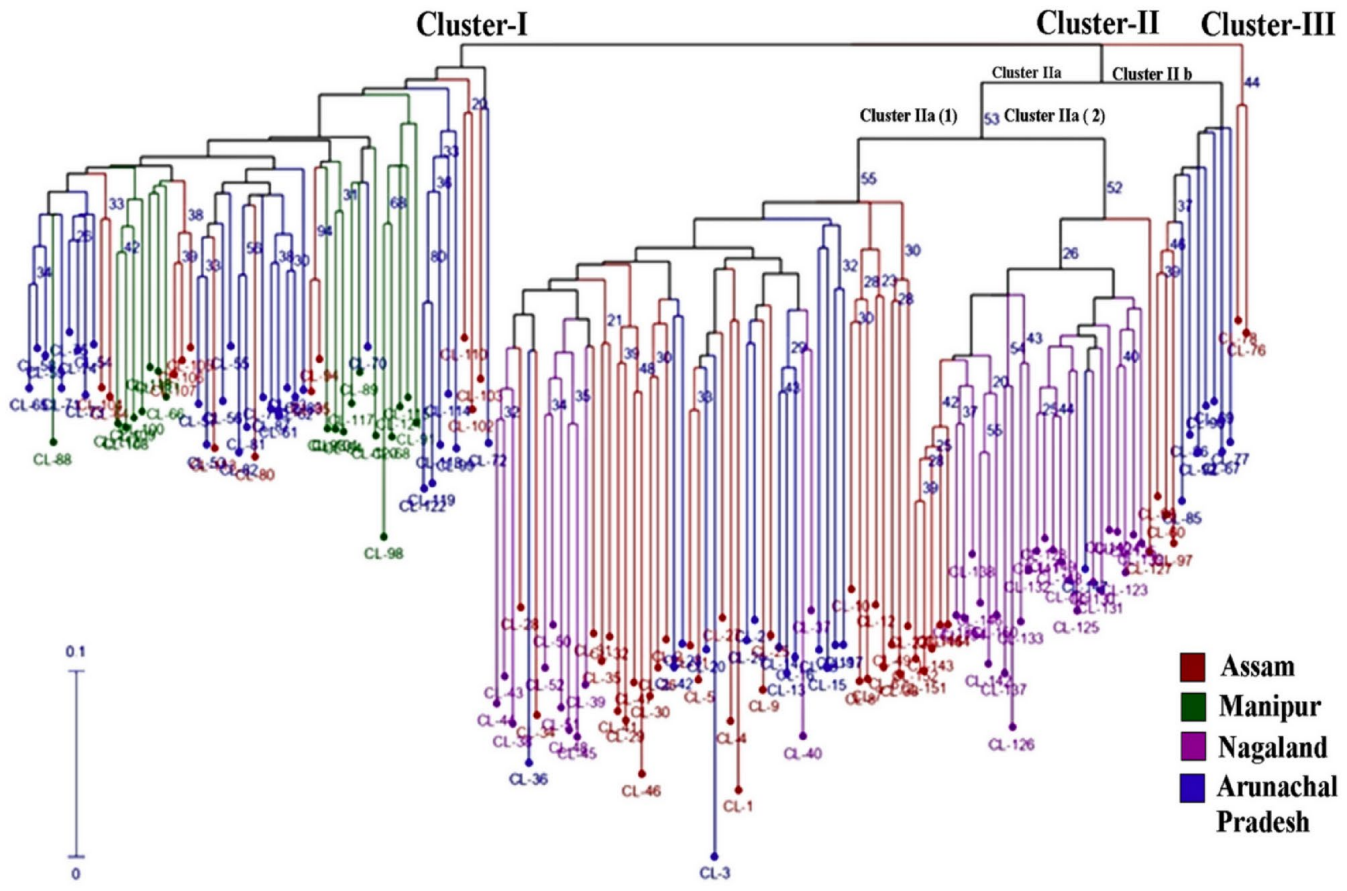
Dendrogram constructed based on N-J method determining three clusters of *C. longa* germplasm; numbers depicted in the clusters represent the code number of 152 accessions.

Neighbor joining method based on Jaccard’s pairwise distance matrix was used to construct the dendrogram of 152 accessions of *C. longa* rich in curcumin content analyzed through SSR marker. A comprehensive study on dendrogram revealed a total of three clusters, out of which cluster I consist of 58 accessions, cluster II consists of 92 accessions, and cluster III consists of 2 accessions which are represented in (Fig. 1). In cluster I, out of 58 accessions, 12 accessions were collected from Assam, 27 from Arunachal Pradesh, and 19 from Manipur. The cluster II is again split up into two sub-clusters: cluster IIa and Cluster IIb where Cluster IIa consists of 82 accessions which are again split up into two minor sub-clusters: cluster IIa(1) consists of 52 accessions gathered from the states of Nagaland (11), Arunachal Pradesh (14), Assam (27) while cluster IIa(2) consist of 30 accessions from the states of Arunachal Pradesh (1), Assam (6), Nagaland (23). Additionally, cluster II(b) consists of 10 accessions comprising from the states of Assam (3) and Arunachal Pradesh (7). Cluster III has a total of 2 accessions (RRLJCL 78 and RRLJCL 76) from Assam.

Jaccard’s Pairwise coefficient outlined maximum genetic dissimilarity of (0.75) between accession RRLJCL 3 of cluster IIa(1) and RRLJCL 126 of cluster IIa(2), indicating high variation between these two lines as it was from two different states viz Arunachal Pradesh and Nagaland respectively and minimum genetic dissimilarity of (0.09) between cluster I of line RRLJCL 58 and RRLJCL 59 indicating significantly less variation as the two lines were from same state, i.e., Arunachal Pradesh.

**Figure 2:**
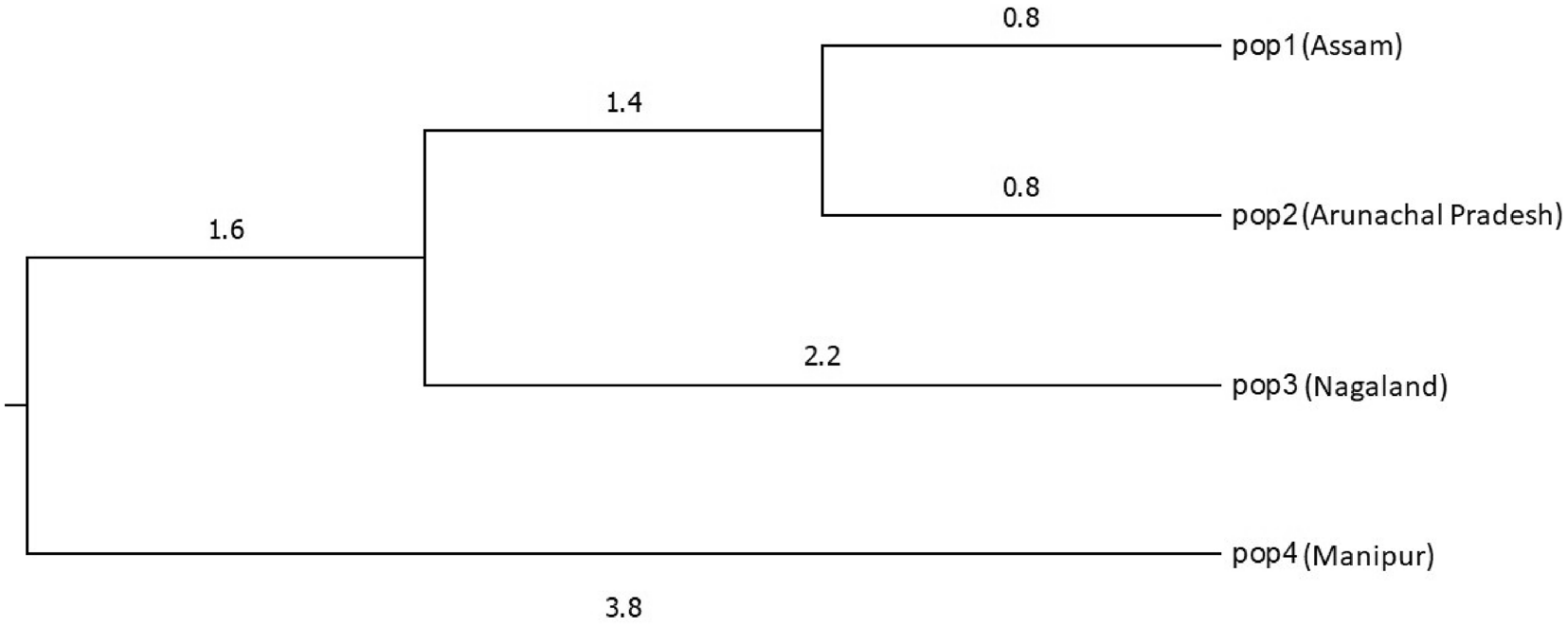
Dendrogram differentiating the genetic divergence among the population based on Nei's Genetic distance: Method = UPGMA.

Based on Nei’s genetic study, genetic distance and genetic identity was calculated between four population, where it was revealed that maximal genetic identity (0.9853) was observed between Pop 1 (Assam) and Pop 2 (Arunachal Pradesh) and maximum genetic distance (0.1220) was noticed between Pop 3 (Nagaland) and Pop 4 (Manipur) which explains that genetic similarity was very high between Pop 1 and Pop 2 whereas genetic variation is high between Pop 3 and Pop 4 (depicted in Table 4; Fig. 2).

**Table 4:** Genetic identity and genetic distance between four populations of *C. longa* based on Nei’s genetic study where Pop 1, 2, 3, 4 represent Assam, Arunachal Pradesh, Nagaland and Manipur respectively.

Pop ID	1	2	3	4
1	****	0.9853	0.9690	0.9338
2	0.0148	****	0.9469	0.9651
3	0.0315	0.0545	****	0.8852
4	0.0685	0.0355	0.1220	****

**Figure 3:**
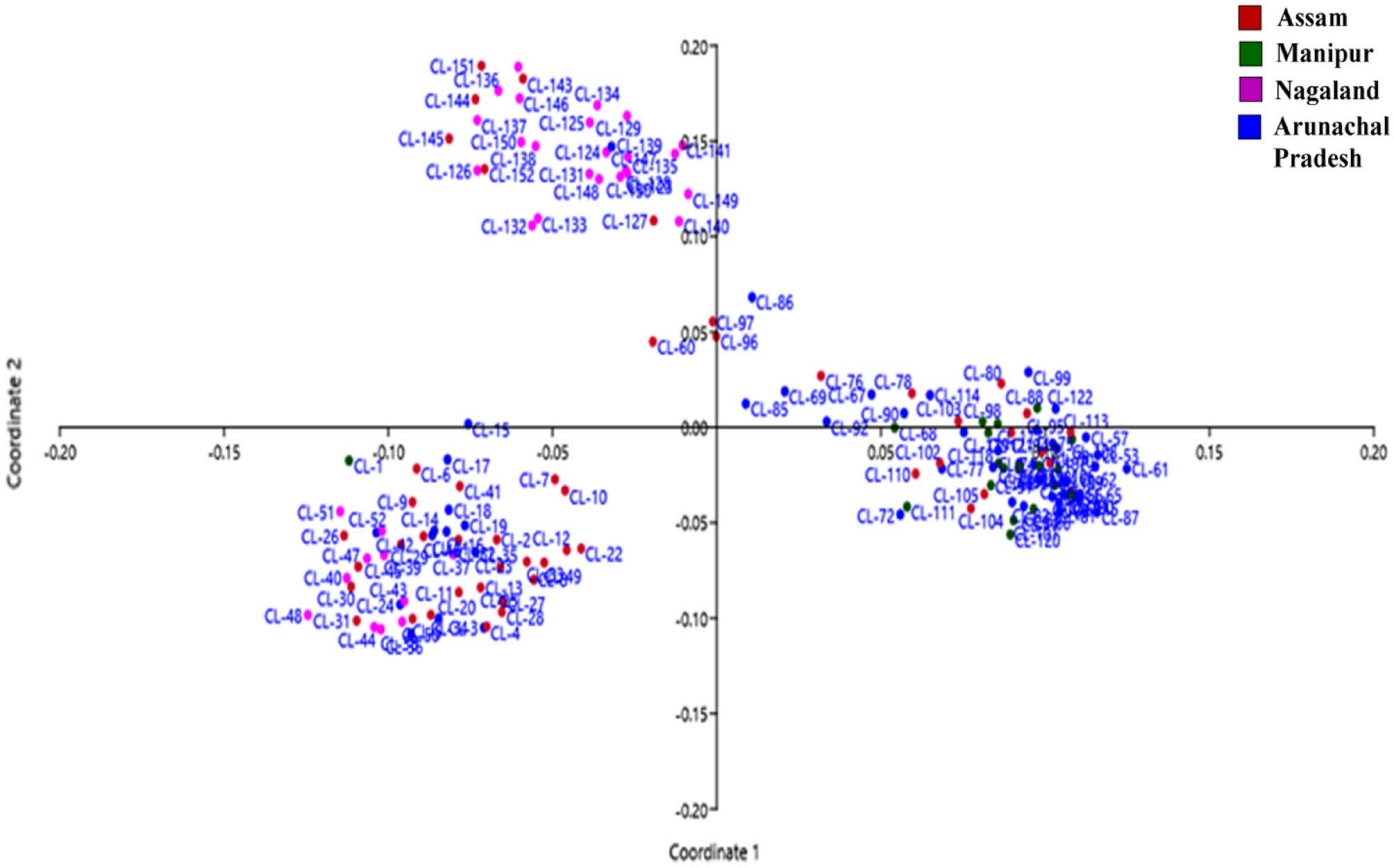
Principal Component Analysis plot of all the accessions of *C. longa* collected from different regions of North East, India.

Principal Component analysis (PCA) was also performed for 152 accessions of *C. longa* to check variability and relationship among them. The highest Eigen value calculated from the first three groups was (3.90, 2.52, 1.34) respectively, which provides more information about the divergence among the accessions. The sum cumulative variance perceived was 34.01%, of which 17.53%, 11.34%, and 6.03% were for the first three principal components (charted in Table 5). The Principal Component Analysis plots coincide mostly with the dendrogram clusters except for a few lines (RRLJCL 60, RRLJCL 86, RRLJCL 96, RRLJCL 97) plotted distance apart, as shown in the PCA plot (Fig. 3).

**Table 5:** Eigen value, percentage variance, cumulative variance of principal component analysis (PCA) on the basis of SSR marker.

Axis	Eigen value	Percent	Cumulative variance
1	3.9063	17.535	17.535
2	2.5279	11.348	28.883
3	1.3441	6.0339	34.0169

### Population structure

**Figure 4:**
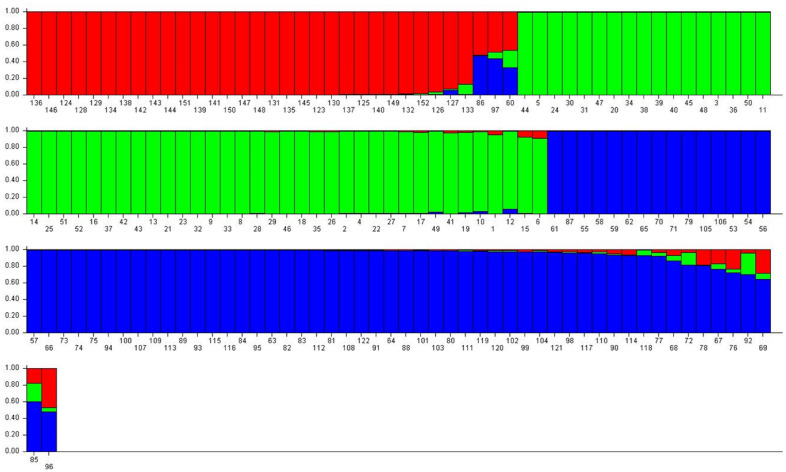
Approximation of relevant number of population in 152 accessions of *C.longa.*

**Figure 5:**
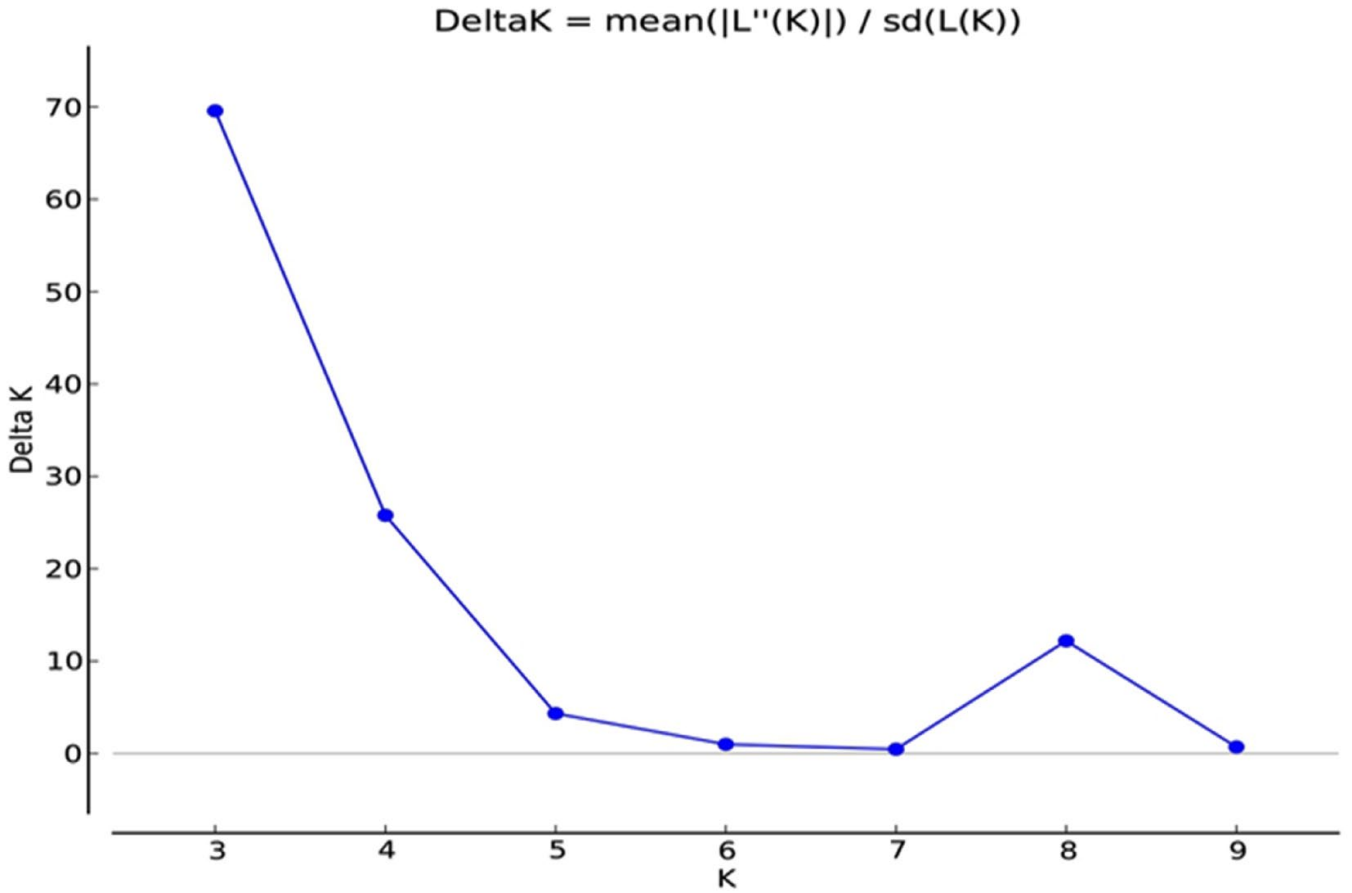
Model-based population structure analysis of *C. longa.*

Based on the structure Harvester software analysis, a total of three subpopulations was constructed, which is shown in (Figs. 4 and 5). Accessions scoring more than 0.80 can be considered as genetically pure accession, and not more than 0.80 can be contemplated as intermixture accession^34^. In this study, almost all accessions were found to be pure accessions except nine accessions which were intermixture in nature. The admixture accession of population structure 1 (red colour) were RRLJCL-86, RRLJCL-97, RRLJCL-60; and RRLJCL-67, RRLJCL-76, RRLJCL-92, RRLJCL-69, RRLJCL-85, RRLJCL-96 were the admixture accession of population structure 3 (blue colour). All the accessions clustered in population structure 2 (green colour) were pure accessions, as shown in (Fig. 4). The Fst mean value of all three populations (1, 2, 3) were given as 0.507, 0.217, and 0.488, respectively. Also, the allele frequency variance between the populations computed using point estimation of P is presented in Table 6.

**Table 6:** Allele frequency divergence among and within the population using point estimation of P.

	1	2	3
1	–	0.0970	0.1263
2	0.0970	–	0.1086
3	0.1263	0.1086	–

### Analysis of molecular variance

**Figure 6:**
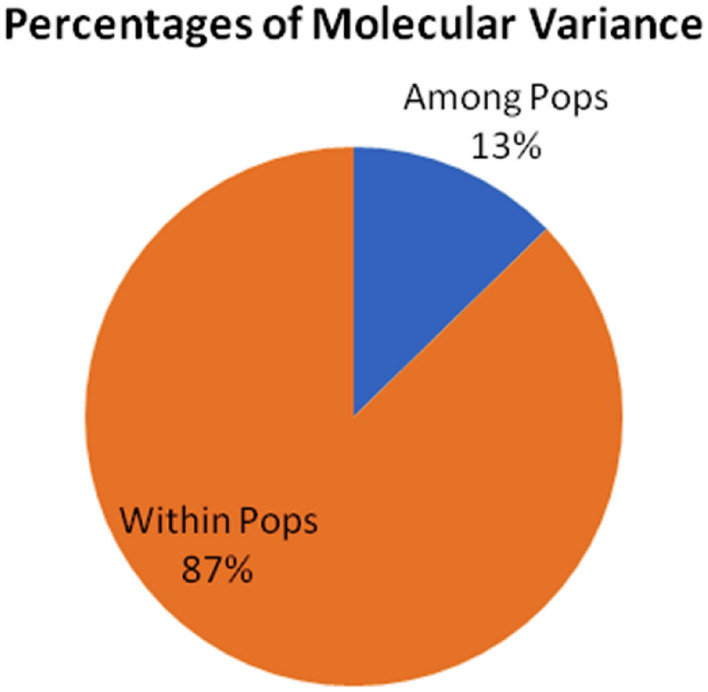
Pie chart of AMOVA (Analysis of Molecular Variance) among and within the population of *C. longa.*

Analysis of Molecular Variance (AMOVA) was conducted for *C. longa* to assess the difference in population, where it was observed that molecular variation was high within the population (87%) and significantly less variation among the population (13%) mentioned in the (Table 7 and Fig. 6).

**Table 7:** AMOVA (Analysis of Molecular Variance) among and within the population of all the accessions of *C. longa.*

Source	Degree of freedom (df)	The sum of squares (SS)	Mean square (MS)	Estimated variance component	Percentage of total variance (%)
Among population	3	368.789	122.930	2.828	13
Within population	148	2876.060	19.433	19.433	87
Total	151	3244.849	142.363	22.261	100

## Discussion

*C. longa* holds industrial significance worldwide and hence needs improvement through breeding program.The collection and assessment of germplasm diversity are imperative to facilitate genetic improvement in the crop. The present study scrutinized high curcumin lines from 329 accessions where 152 accessions showed curcumin content > 1.50%. Among the 152 accessions, 59 accessions showed curcuminoid content > 9%, out of which 29 accessions were from the state of Assam, 18 accessions were from Arunachal Pradesh, 10 accessions were from Nagaland and 2 accessions were from Manipur. The elevated curcumin content was observed mostly in the accessions grown in Assam and Arunachal Pradesh which can be attributed to the favorable environmental conditions in Assam as the state shares a moderate climate condition, characterized by an average temperature of 25.67 °C and an average humidity of 75.15% with an annual rainfall of 2244 mm. This aligns with the findings of Sandeep et al.^39^ who also noted that the curcumin content in turmeric is influenced by the specific environmental zones in which the plants are cultivated. Thus, it can be stated that soil and environmental factors plays a significant role in the accumulation of curcumin in turmeric plants.

Keeping in view the effect of environmental factors on the genotypes of *C. longa* which hinders the phenotypic attribute in germplasm variation, a molecular diversity study on 152 high curcumin accessions were conducted using SSR markers. The molecular markers or primers serve as indicators of polymorphism in unveiling genetic distinctions among individual organisms or species which may emerge from its genetic constitution, nucleotide alterations or genome locus mutations^40^. A total of 39 SSR primers were screened, where all the primers showed good amplified bands with 100% polymorphic percentage. Likewise, Singh et al.^37^ studied 9 SSR primers on 30 genotypes, out of which only 6 primers pairs showed 100% polymorphism with banding patterns ranging between 1–2 allele/loci. A study on 60 genotypes of turmeric was reported where average polymorphism resulted 91.4% and 95.4%, respectively, for 11 RAPD and 6 ISSR primers^41^. Again 15 DAMD and 13 ISSR primers were studied by Verma et al.^42^ where the average polymorphism percentage reported was 84.4% and 79.2%, respectively; Sumi et al.^43^ reported 64.4% average polymorphism percentage on 8 genotypes by using 12 RAPD primers. Aswasthi et al.^44^ also worked on 56 primers which include both SSR and ISSR on 18 improved varieties. Similarly, in the present survey, all the screened 39 primers represent a high level of polymorphism percentage (100%) as compared to earlier studies, which outlines an indication towards high level of genetic variation among the individual accessions. In addition to that, PIC, MI, and Rp values were evaluated where the PIC value for all the primers ranged between 0.20 and 0.89, where MAGN 27 showed minimum PIC value, and CuMiSat 31 showed maximum PIC value. The average PIC value obtained from 39 primers is 0.64, out of which 31 primers showed a PIC value higher than 0.50 signifying that SSR primers used in this study are highly informative as PIC value greater than 0.50 (> 0.50) illustrate high efficiency of primers^34,45^. The primer proficiency was further analyzed through MI and Rp where the average MI was 2.82, with the lowest value showed by primer (MAGN 27) and the highest value showed by primer (CuMiSat 22). Again, average Rp was calculated to be 1.204, where the lowest value showed by primer (RM 171), and the highest value showed by primer (CuMiSat 37). Based on study and observation of data for all the screened primers, eleven primers, namely CuMiSat-19, CuMiSat-21, CuMiSat-22, CuMiSat-31, CuMiSat-35, RM125, RM135, RM153, RM154, CBT-05, and CBT-08 showed most efficient outcome with PIC, Rp and MI values more than the mean values (PIC = 0.64, MI = 2.82 and Rp = 1.20).The findings of the above analysis unveiled efficiency of the primers which will serve as a resource for future conductance of molecular diversity analysis in different species of Zingiberaceae family.

The genetic diversity analysis led to the construction of cluster dendrogram using Neighbor-Joining (N-J) method as it is a rapid tool and reliable in nature^46^. The N-J method is a distance-based method that relies on assessing shared alleles to calculate distances between taxa, creating distance relationships of different accessions or germplasms in representation of tree^47^. In the present investigation, the results obtained through cluster dendrogram revealed the presence of geographically independent clusters as majority of the accessions did not exclusively grouped according to geographical provinces, and the reason may be due to the migration of genotypes from one location to another caused by overexploitation of natural habitat^34,48^. However, region-specific clustering was restricted to cluster III, where 2 lines (RRLJCL78 AND RRLJCL76) were from Assam, and the reason may be due to uninterrupted wild varieties in natural habitat^1^. Likewise, Verma et al.^42^ identified two prime clusters from the dendrogram constructed on account of the combination of DAMD and ISSR markers, where it was revealed that genotypes in the clusters grouped independently of their geographical location which align with our study. Again, Sahoo et al.^49^ worked on 10 Curcuma species using EST SSR primer, revealed the presence of two main clusters, one of which consists of all nine species and the other of only *Curcuma longa* species. The clustering of accessions in different clusters unveiled more genetic distance, revealing more genetic variation which would be conducive to the future hybridization programme. The genetic diversity analysis of intra and inter-population genotypes were also studied in the four populations which was divided based on its collection site. All the populations showed good polymorphism, with only moderate polymorphism in the Manipur population, which may be due to the low number of accessions collected from Manipur compared to other populations used in the study. All the genetic parameters for diversity revealed highest variation in population 1 (Assam) and lowest in population 4 (Manipur). Here, the reason for highest variation in population 1 may be due to the exchange of accessions from the neighboring states or random mutation. Moreover, significant genetic diversity was confirmed from the results of total species diversity within the population (Ht) (0.27 ± 0.03) and among the population (Hs) (0.23 ± 0.02), with genetic differentiation degree (Gst) of 1.24. The parameter like gene flow (Nm) is the shifting of genetic distinction from one population to another, and when gene flow increases among populations, it indicates less variation and more homogeneity whereas when gene flow increases within population, it indicates high variation and less homogeneity. In this study, gene flow (Nm) was found to be 3.51, which is significantly higher than the threshold value (Nm ≤ 1.0)^50^, and represents high gene flow, whereas Sahoo et al.^49^ reported working on genetic variation of Curcuma species using EST SSR primer where Nm value observed was 0.0781 which revealed that whole distribution is highly constricted and genetic distinction is highly noteworthy. Again, Basak et al.^51^ while working on turmeric cultivars using ISSR and RAPD markers reported the Nm value to be between 0.33 and 0.37 for both markers. Here the present study revealed moderate genetic heterozygosity and genetic differentiation degree, which determine moderate genetic variation among and within the populations, and it can be concluded in context to the previous reports^34,52^. The results in gene exchange in the present study may be due to human intrusion like genetic swamping, introgression and hybridization^1^.

Through Nei’s genetic study, genetic identity among the population ranges from 0.9853 to 0.1220 with maximal genetic identity of 0.9853 was found between Pop 1 (Assam) and Pop 2 (Arunachal Pradesh) and the minimal genetic identity of 0.1220 was found between Pop 3 (Nagaland) and Pop 4 (Manipur), and the reason for low variation, i.e., maximal genetic identity between Assam and Arunachal Pradesh population may be due to gene exchange or duplication of germplasm between adjacent states as gene exchange among two population leads to decrease of genetic variation and increase of homogeneity^53^. Similarly, Nei’s genetic study on *Cymbopogon winterianus* was studied by Munda et al.,^34^ where low variation was found among the population and mentioned that the reason for low genetic distance was due to gene exchange among the adjoining population. Moreover may have occurred through human intrusion since gene exchange cannot be facilitated through pollen or seed because the showy flower which blooms in *C. longa* plant is not a true flower but is actually a bract. Thus, the data procured through this study could be used as baseline data for selection of desired traits in breeding programmes and conservation of highly variable germplasm^54^.

In addition to that, Principal Component Analysis (PCA) is broadly used to study the structure of data^55^, where PCA is useful in clustering of similar accession while non-similar accession plot distance apart in the presentation^56^. In the present survey, the PCA plots closely align with the dendrogram clusters, except for a few outliers (RRLJCL 60, RRLJCL 86, RRLJCL 96, RRLJCL 97) that are plotted at a significant distance from the main cluster. The PCA and cluster analysis provide more reliable information if the first three component scores more than 25% variance^57^. In the present study, the first three components (17.53%, 11.34%, and 6.03%) come up with 34.01% variability, which is higher than previous reports and indicates more reliable data. Unfortunately, there is currently limited data available regarding the intra and inter-specific relationships and genetic diversity within large number of germplasm of *C. longa* collected from North East India. Thus, this study will contribute a novel insight to researchers and breeders towards development of superior variety.

Further, the structure analysis employs a Bayesian clustering approach, assigning individuals to grouped as populations based on their genotypes and seeking population structures characterized by linkage equilibrium and Hardy–Weinberg equilibrium^47^. From the population structure survey, a total of three population structure were constructed where population 1 is represented with red colour, population 2 is represented with green colour and population 3 is represented with blue colour. As accessions scoring more than 0.80 can be considered as pure accessions, here in the present analysis, almost all accessions were found to be pure accession except nine accessions (RRLJCL-86, RRLJCL-97, RRLJCL-60, RRLJCL-67, RRLJCL-76, RRLJCL-92, RRLJCL-69, RRLJCL-85, RRLJCL-96) were found intermixture in nature. The admixture accessions (RRLJCL-86, RRLJCL-97, RRLJCL-60) were found in the population structure 1 (red colour) and the admixture accessions (RRLJCL-67, RRLJCL-76, RRLJCL-92, RRLJCL-69, RRLJCL-85, RRLJCL-96) were found in the population structure 3 (blue colour) and remaining all the accessions were found to be pure accessions.In the population structure 2 (green colour), no intermixture accessions were found. The pure accessions which were retrieved from this study could be used for conservation of gene pool due to distinct genetic makeup for longer survival of the turmeric species, and this could be later applied for cross breeding programme for development of superior genotype. Through this study, it can be concluded that the SSR markers used in this survey for the genetic divergence of *C. longa* accessions is suitable and satisfactory for population studies. The reason for the genetic divergence or intermixture accessions found within the populations is mainly due to several factors like gene flow, mating process, selection, mutation^1^. Moreover, accessions grouping from bar plot resembled mostly with dendrogram clusters in the current survey which provide more reliability and satisfactory result towards genetic diversity studies. Thus, the neighbor-joining tree and Structure analysis of the SSR data complement each other, offer distinct methodologies for investigating the relationships among different accessions and together yield a robust analysis.

Furthermore, AMOVA study revealed high molecular variation within the population (87%) and significantly less variation among the population (13%). Similarly, Sigrist et al.^26^ also performed genetic divergence of turmeric using microsatellites where genetic divergence within the population was 75.29% and among the population was 24.71% which belongs only to Brazilian states. In contrast, when the accessions were collected from different countries like Brazil, India, and Puerto Rico, the genetic variability was observed to be 63.42% among the countries and 27.05% within the countries. Singh et al.^41^ also conducted the analysis of molecular variance using the RAPD marker, which showed 42% genetic variability among the population, and the remaining 58% was within the population, whereas for the ISSR marker, 48% showed genetic variability among the population and 52% was within the population. Similarly, in present investigation, molecular variation within the population was notably more extensive than among populations, serving as the primary source of overall genetic variation. The lower diversity among populations suggested a heightened level of gene exchange and the higher diversity within the population may be due to random drift, mutation, transgressive segregation^58^ or collection of all the accessions from adjoining states of India which is in accordance to Sigrist et al.^26^ study. As per the genetic theory of populations, an increase in diversity enhances a species potential to adapt to evolving environments. Hence it becomes necessary to delve into knowledge of genetics, as the loss of heterogeneity could risk the population's sustainability and ultimately lead to the extinction of the species^1^.

To devise effective conservation strategies for various species, understanding the genetic makeup within populations is of utmost significance and to prevent the depletion of genetic foundations, selection of genetically diverse genotypes, analysis of plant molecular diversity is an indispensable tool^54,59^. From an evolutionary perspective and for the long-term survival of species, population genetic diversity of *C. longa* holds immense importance. Therefore, through this genetic assessment study, eleven most efficient primers were found which showed PIC, Rp and MI values greater than the average PIC, Rp and MI values. A total of three clusters were formed through Neighbour Joining dendrogram, structure bar plot and PCA analysis which resembles with each other. Analysis of variance revealed high molecular variation within the population (87%) and very less variation among the population (13%) and maximal intra-population diversity was distinguished in Pop 1 (Assam) and minimal in Pop 4 (Manipur). Hence it is necessary to conserve the highly variable population through both *in-situ* and *ex-situ* conservation, which may otherwise lead to a loss of heterogeneity in a population, causing species extermination. In the present study, population 1 (Assam)which showed highest variation can be invaluable for hybridization efforts in future crop improvement programs and the identification of closely related species which are represented as pure accessions can serve as major sources of genes for conservation of gene pool and future breeding programs. Therefore, result obtained through genetic assessment study of *Curcuma longa* L. will provide a handful of resources and great potential for enhancing crop improvement programs, facilitating the selection of desired genotypes, aiding in marker-assisted breeding, identifying quantitative trait loci, and preserving the genetic diversity. The growing global demand for curcumin, the most active component found in turmeric, due to its numerous health benefits underscores the importance of conserving and cultivating curcumin-rich superior genotype and hence urgent steps must be taken to effectively conserve and breed these industrially significant plants. Hence, the present research report will play a pivotal role in promoting the sustainable growth of this economically important medicinal species.

## Methods

### Plantation of collected germplasm of *C. longa*

**Figure 7:**
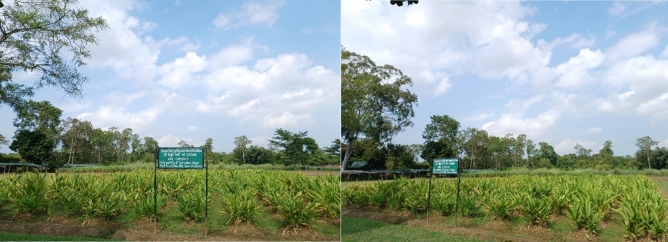
Experimental trial of 329 accessions of *Curcuma longa* L. collected from four states of North East India.

A sum of 329 accessions of *C. longa* was collected from four states of India, specifically from Assam, Nagaland, Arunachal Pradesh, and Manipur during the year 2019 (presented in Fig. 7). The sample specimens were identified by Dr. Mohan Lal, Principal Scientist, CSIR-NEIST, Jorhat. The specimen herbariums with voucher no. RRLJCL-1 to RRLJCL-152 were deposited at the departmental herbarium. The collected rhizome was then planted in augmented design during the month of March 2020 at the Experimental farm of CSIR-NEIST Jorhat. The experimental site received an annual rainfall of 2244 mm, a mean temperature of 25.67 °C, and mean humidity of 75.15%. The soil pH was 4.9, sandy loam in nature, and NPK concentration was nourished at 226, 116, and 144 kg/ha, respectively. The spacing of one plant from another plant was maintained at 45 cm, and row-to-row spacing was also 45 cm. After harvesting, each germplasm rhizome (300 gm) was dried for 7 days, and the curcuminoid content was measured for each. Among them, 152 high curcumin content (> 1.5%) lines were selected and again planted in Randomized Block Design (RBD) with three replications during the month of March 2021, which were used for molecular diversity assessment. All the experimental research and field studies performed on 329 accessions of *Curcuma longa* L. were carried out in accordance with relevant guidelines.

### High curcumin lines selection from collected germplasm

All the planted accessions were estimated for their total curcuminoids content as per the below-described procedure. Firstly the fresh rhizomes were cut into small pieces and dried in a hot air oven for seven days at 59 ° C and then were grind to make fine powder. The fine powder sample was further used for solvent extraction with acetone (100 mL) using the Soxhlet apparatus. The solvent extract was allowed to evaporate through the rotary evaporator to obtain the dry mass. The dry mass was put forwarded for HPLC analysis where a Thermo Scientific Dionex Ultimate 3000 HPLC system was used with Syncronis C-18 column (Dim. 150 × 4.6 (mm), SN: 10,879,123, LOT: 16,092, Particle size (µ) 3), acetonitrile and 0.1% formic acid as mobile phase, the flow rate of 1 mL/min, injection volume of 20 µL, UV: 426 nm, temperature: 25ºC as conditions. For the development of a standard calibration curve, 1 mg of standard total curcuminoids batch number ANE/CL/01/047/21 (purchased from Indica Neutraceuticals, India) was dissolved in 1 mL of HPLC grade acetonitrile from which different concentrations were prepared (20, 40, 60, 80 and 100 ppm) and injected into the system. Samples were prepared by using 1 mg of dried extract in 1 ml of HPLC grade acetonitrile, filtered with 0.2 μm nylon membrane, and injected into the system. Chromatographic data were obtained from which data were analyzed using Thermo Scientific Dionex Chromeleon Chromatography Data System version 7.2.

### Extraction of genomic DNA from *C. longa* accessions

Fresh tender leaves of all the selected high curcumin-rich accessions (152 no.) of *Curcuma longa* were collected in separate zip lock bags from the experimental field at CSIR NEIST, Jorhat, Assam. Leaf samples were then washed, cleaned properly, and lyophilized at − 40 °C for 48 h. Isolation of plant DNA was carried out using HiMedia Kit (HiMedia Mumbai) by modifying the CTAB method (Cetyltrimethylammonium Bromide) as instructed in the kit. Agarose gel 0.8% was used to check the purity of extracted DNA, and bands were observed in the gel documentation system (Vilber E-Box, France). To check the quantification of stock DNA concentration, 3 µl of DNA sample was assessed in a Nano Bio Spectrophotometer (Eppendorf, Germany) at λ^260^/λ^280^ ratio.

### PCR analysis of *C. longa* accessions using SSR Primer

PCR analysis of *C. longa* accession outlined 39 primer pairs out of a sum of 44 pairs of SSR primers which showed the best amplification for genetic study analysis. The primers were selected based on the earlier literatures on molecular diversity analysis in the Zingiberaceae family, which was then obtained from Bioserve Biotechnologies (India) Private Limited, Hyderabad, India. The primers obtained were in lyophilized form, which was then prepared to stock solution followed by preparation of working solution at a concentration of 10 picomol. The extracted DNA of all the accessions was also converted to a working solution with a concentration of 30 ng. The required mixture for PCR amplification consists of 5μL of DNA sample, 1μL each of forward and reverse primer, 10 μL of master mix (HiChrome), and 3μL of doubled distilled water, making a final volume of 20μL. For amplification, Prima 96 thermocycler (HiMedia, India) was used, which was conditioned at 95 °C for 1 min for initial denaturation, then continued with 35 cycles of 95 °C for 55 s (denaturation), 55 s with primer melting temperature ± 5 °C (annealing), 72 °C for 5 min (extension), and finally by 72 °C for 8 min as a concluding extension. The amplified results were then observed in Agarose gel (2%) using 1X TBE buffer, and electrophoresis was run for 1 h at 90 constant voltage. The amplified bands were then observed in a gel documentation system (Vilber E-Box, France).

### Statistical data analysis

In order to obtain genetic variation, the banding patterns were scored after observing in the gel documentation system. The data was obtained by using input as 1 for the presence of band and 0 for the absence of band for each primer in the genotypes. The Jaccard's similarity coefficient was evaluated to obtain all pairwise differences between the accessions to build a genetic dissimilarity matrix. The final cluster dendrogram and principal coordinate analysis (PCA) were plotted based on the dissimilarity matrix and Neighbor Joining (N-J) method using Darwin software version 6.0.

Polymorphic Information Content (PIC) values were calculated by using the formula PIC = 1 − ${\sum pi}^{2}$ on the basis of the polymorphism of the bands where Pi = frequency of ith allele^45^. Marker Index (MI) was calculated by the formula MI = EMR × PIC where EMR is the Effective Multiplex Ratio and is defined by the multiplication of a number and a fraction of polymorphic loci^60,61^ while Resolving Power (Rp) is the summation of the band informativeness calculated by the formula [I_b_ = 1 − [2 ×|0.5 − P|] where p = proportion of the individuals containing the bands^61^. In addition, the POPGENE (Version-1.31) software was utilized to study the genetic diversity variables such as genetic differentiation degree (Gst), genetic diversity in the population (Ht), gene flow (Nm), genetic diversity within the population (Hs), Shannon’s information index (I), number of observed alleles (na), number of effective alleles (ne) and Nei’s gene diversity (h). Through Analysis of Molecular Variance (AMOVA), inter and intra-population diversities were analyzed by implementing GenAlex software version 6.5. To evaluate the genetic relationship among 152 accessions using 39 SSR primer STRUCTURE software version 2.3.4 has been utilized to construct a model-based population structure where the software was pass multiple times to evaluate the number of populations among all the accession. The most foreseeable genetic population group obtained from the study was computed by using an online tool named Structure Harvester.

### Supplementary Information

Supplementary Figure 1.

## Data Availability

The datasets used and/or analysed during the current study would be available from the corresponding author on reasonable request.
