# Supplementary figures and images for: Molecular genetic divergence analysis amongst high curcumin lines of Golden Crop (Curcuma longa L.) using SSR marker and use in trait-specific breeding
Source: Sci Rep. 2023 Nov 11;13:19690. doi: 10.1038/s41598-023-46779-5 (PMC10640617; doi:10.1038/s41598-023-46779-5)

**
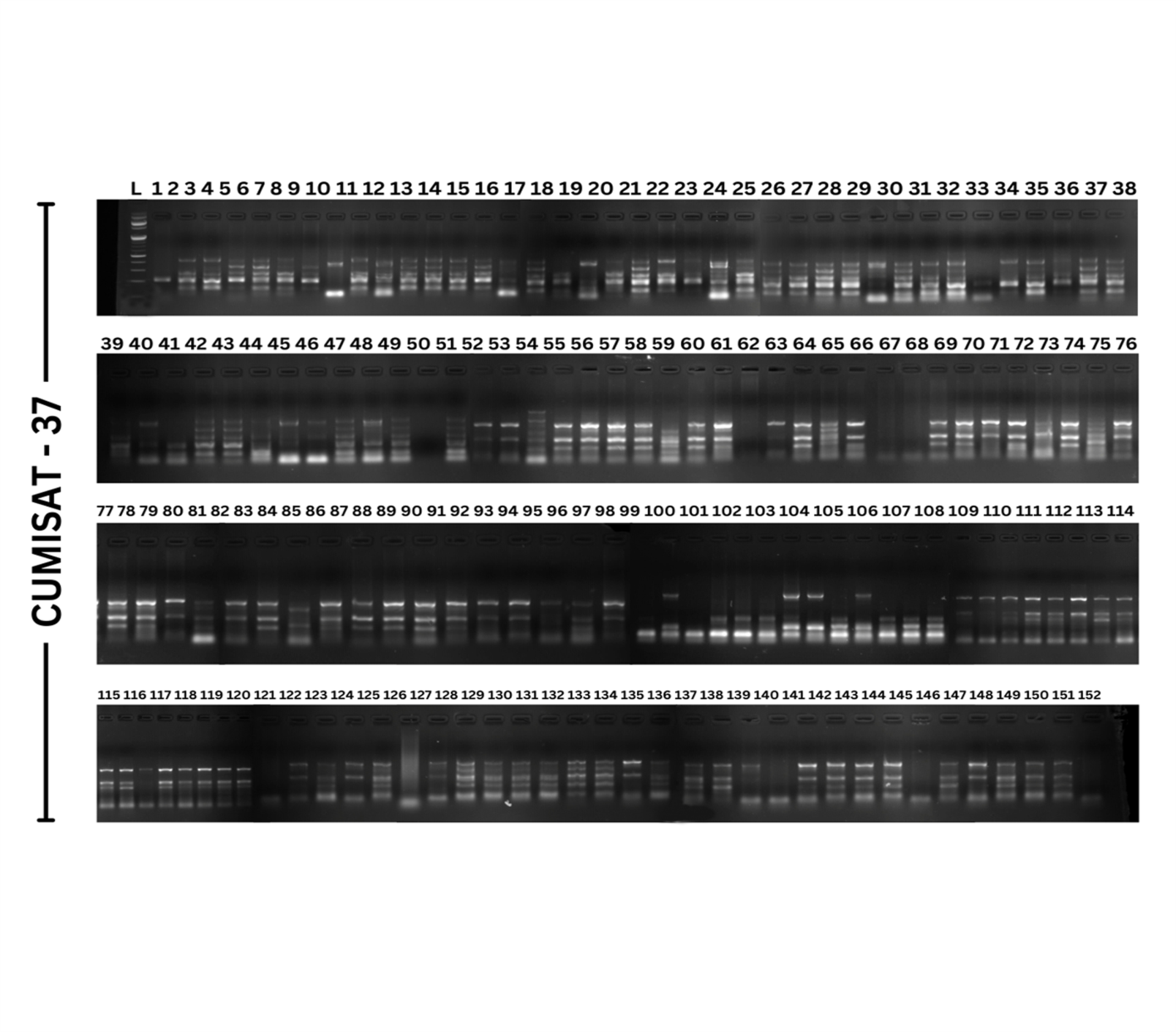
**

**Figure S1.** Gel image of SSR primer (CuMiSat 37) obtained from 152 germplasm of *Curcuma longa*

Supplement: Supplementary file 1 — Supplementary Figure 1. [file 41598_2023_46779_MOESM1_ESM.docx]
